# Surgical Drainage

**Published:** 1910-05

**Authors:** L. Sexton

**Affiliations:** New Orleans, La.


For Texas Medical Journal.

### Surgical Drainage.

BY L. SEXTON, M. D., NEW ORLEANS, LA.

Surgical drainage is the mechanical method of keeping abscess cavities emptied, infecting substances and wound secretions removed. This exudation in fresh operations or wounds is serous at first, but may become seropurulent and finally pus if infected.

Small clean wounds and large surgical or accidental incised wounds, if not infected, should be closed at once without drainage.

Drainage is usually required wherever the deep aponeurotic structures and muscular sheaths or joint cavities have been opened, where much blood, or large effusions are present after removing cysts; in urinary and gall-bladder operations in the larger amputations, as of the breast and thigh, and after operating on neck and axillary lymphatic glands.

Again, all extensively lacerated and comminuted wounds likely to become infected had best be left open or loosely packed with gauze, which gauze may be saturated with some antiseptic solution if infection is supposed to already exist in the wound.

All large sized surgical or accidental wounds, with much effusion or hemorrhage, should have a small piece of drainage gauze left in the most dependent portion, usually for twenty-four hours. The end of this gauze may be left protruding from under the dressing so that it may be easily removed without disturbing the superimposed absorbing material. Whenever infection is present at the inception of the wound or likely to occur afterwards, it is best to pack with sterilized gauze and to leave the wound open and sutures untied, which sutures have been introduced at the first dressing of the case. Large non-operative wounds should be subjected to frequent inspection, and at the first approach of sepsis all sutures should be removed, and the open surfaces of the wound flooded with sterilized saline solution to facilitate drainage and the removal of secretions.

Deep incised or punctured wounds should usually have their openings enlarged and some kind of drainage inserted.

It should not be neglected to enlarge such wounds with small external openings to better facilitate the discharge of any serum or infection which might otherwise be pent up in the closed wound. In all injuries to subcutaneous tissue with a large hematoma likely to follow, it is prudent to put a stab wound into the most dependent portion to drain off the exudate, thus obviating all dead spaces which are but hotbeds for germ development.

Drainage material is usually composed of sterile or medicated gauze, silk worm, horse hair or catgut, rubber tissue, strips of oil silk (the latter substance having the advantage of being easily removed). Sterilized rubber tissue, with gauze longer than the tissue, with alternate layers of the gauze and rubber tissue, makes a splendid cigarette drain. Oil silk or rubber tissue may be perforated and wrapped around gauze drainage material for deep wounds into the abdominal cavity, thus facilitating their easy removal and preventing their adhesion to the peritoneum. Rubber and glass tube drains are suitable where the pus is thick and discharge abundant and likely to last a considerable length of time. The rubber tube has the advantage over the glass tube, in that its length can be regulated by cutting it off to suit the depth of the wound, but the glass tube has the advantage of being more cleanly and less likely to become infected. The glass and rubber tubes are removed and gauze substituted for them as soon as possible in order to prevent the likelihood of hernia following the wound, also to promote the healing, as these hard tubes damage the granulation by pressure; they may also cause fistula to form, and in some instances have been known to slough the bowels from continued pressure. The hard glass and stiff rubber tubes act more as foreign bodies in such cases than does the more pliant antiseptic gauze dressing.

While exact rules are impossible to record in regard to drainage, because no two cases are exactly the same, the patient's environments and resistance has something to do with the kind of drainage it is best to adopt. Occasional operators need to use drainage oftener than surgeons who are constantly operating and whose technique and material is less likely to be followed by sepsis. Again, it should be remembered that in large lacerated wounds, produced accidentally, they are usually infected at the very start, though they may not necessarily suppurate if properly treated at the first dressing.

In cases of empyema, abscess of the liver, gall and urinary bladder, drainage tubes are very much preferable to gauze. It should always be remembered that gauze soon becomes clogged with fibrine and does not drain pus at any time. An objection to absorbable material is that we may infect the wound that we are trying to drain with it.

Gauze only drains serum, and that for a few hours only. We must never forget that lives may become imperiled by leaving large amounts of gauze in the abdominal cavity for too long a time as drainage.

In all large appendiceal abscesses it is well to wall off the wound with gauze for a time, for by so doing (as pus will not go through the gauze) we may prevent general peritoneal infection. We must also remember that irrigating wounds too often may furnish an entrance for bacteria, which we sometimes attribute to the drainage producing the infection. Frequent dressings in such cases is imperative.

Wherever there is pus evacuate it is an axiom in surgery which might be applied as well to blood and serum in newly-made wounds as to pus in abscess cavities. Drainage, therefore, in the direction of least resistance is one of the most important aids to successful surgery. In any operation where we are not absolutely sure that there would be no oozing or secondary hemorrhage, drainage should be provided for. It is equally important in compound fractures, and in operations where the scrotum is involved, or wherever in operating a cavity or dead space is left which might fill with blood clots or serum. In lacerated gunshot wounds and violently infected tissue. If any of these traumatic conditions exist over bony prominences where pressure can be exerted upon the underlying bony structure, the drainage is not so essential as the oozing under such conditions may be controlled by compresses and bandaging rendering the use of drainage unnecessary. Since the adoption of drainage in appendiceal operations, where large pockets of the peritoneal cavity is more or less filled with pus, mortality has been diminished 75 per cent.

We are not content in cases just enumerated with a drain in the lower edge of the incision, but stabbed wounds are made in the abdomen in the opposite side, and very often through the lumbar region as well, so that no pus may be pent up to cause fever. Some of the older surgeons who adopted this plan of lumbar drainage in appendiceal abscess before the actual cause of the abscess was known had a large number of recoveries resulting from this through and through drainage into the most dependent portion of the appendiceal abscess, which, as a matter of course, had been previously walled off by peritoneal adhesion. The appendix in such cases undergoes a molecular death passing away in the pus which is so freely evacuated through this extensive opening. Gynecologists have learned to utilize the benefits of drainage through Douglas' cul-de-sac in the majority of pelvic abscesses, whether resulting from gonococci infection or that following abortions, and other pelvic inflammations, and while it is not our intention to discuss this phase of surgical drainage, the mortality from such conditions has been greatly reduced since the rule of free excavation and packing of the pelvic cavity with gauze has been a recognized treatment. In amputations of the thigh or breast where large raw surfaces are necessarily produced a small strip of gauze left in the most dependent portion of the incision for twenty-four or forty-eight hours is the saving feature and great promoter of union by first intention by permitting these exudates to drain out into the dressings rather than to form dead spaces of culture fluids in the traumatized area.

As a matter of course, in ordinary abdominal operations where no pus cavities are encountered, and where no extensive inflammatory adhesions have been broken up, it is much safer to close up these peritoneal and abdominal wounds, but in cases where a large parovarian cyst has been enucleated some provision must be made for twenty-four to forty-eight hours drainage from the considerable amount of effusion which follows such work. We all understand, as a matter of course, that whenever drainage tubes are kept in for a great length of time that the risk of hernia is increased, also that drainage tubes kept in too long may act as foreign bodies, thus keeping up instead of diminishing the discharge of pus. If it is an ordinary effusion that is to be drained, a small strip of sterile gauze is sufficient, and preferable because blood and serum by capillary attraction are evacuated in this way, at least for a while; whereas, the large glass and rubber tubes which are necessary in thick pus cases might do harm instead of good. The perforated drainage tube of rubber and glass are very often occluded by the blood clot or their perforations may become blocked by folds of the intestines.

The criticism of the glass drainage tube in non-suppurative cases is that it fails to adapt itself to the sinuosities of the soft parts, and is unyielding in its position. In draining amputations where we do not wish to disturb the dressing for a week or more, the drainage of the stump may be accomplished, as said before, by coarse absorbable catgut ligatures, or with silk worm and horse hair left long enough to extend out from under the bandage so that they may be removed without disturbing the other dressings in the case.

In dealing with pus cases, drainage of either rubber or glass tubes are preferable.

Surgical drainage is indicated in operations for stone in the bladder, violent cystitis, in large prostate, in gall-bladder infections, in empyema, in pyonephritis and in ulcerated and carcinomatous conditions of the stomach, and in many other conditions which the brevity of this paper will not permit us to include.

